# Deep learning-based image classification for AI-assisted integration of pathology and radiology in medical imaging

**DOI:** 10.3389/fmed.2025.1574514

**Published:** 2025-06-02

**Authors:** Lanting He, Lan Luan, Dan Hu

**Affiliations:** ^1^School of Optoelectronics, Beijing Institute of Technology, Beijing, China; ^2^College of Computer and Information Engineering, Guizhou University of Commerce, Guiyang, China; ^3^School of Big Data and Information Engineering, Guizhou University, Guiyang, China

**Keywords:** medical imaging, deep learning, multi-modal integration, domain adaptation, interpretability

## Abstract

**Introduction:**

The integration of pathology and radiology through artificial intelligence (AI) represents a groundbreaking advancement in medical imaging, providing a powerful tool for accurate diagnostics and the optimization of clinical workflows. Traditional image classification methods encounter substantial challenges due to the inherent complexity and heterogeneity of medical imaging datasets, which include multi-modal data sources, imbalanced class distributions, and the critical need for interpretability in clinical decision-making.

**Methods:**

Addressing these limitations, this study introduces an innovative deep learning-based framework tailored for AI-assisted medical imaging tasks. It incorporates two novel components: the Adaptive Multi-Resolution Imaging Network (AMRI-Net) and the Explainable Domain-Adaptive Learning (EDAL) strategy. AMRI-Net enhances diagnostic accuracy by leveraging multi-resolution feature extraction, attention-guided fusion mechanisms, and task-specific decoders, allowing the model to accurately identify both detailed and overarching patterns across various imaging techniques, such as X-rays, CT, and MRI scans. EDAL significantly improves domain generalizability through advanced domain alignment techniques while integrating uncertainty-aware learning to prioritize high-confidence predictions. It employs attention-based interpretability tools to highlight critical image regions, improving transparency and clinical trust in AI-driven diagnoses.

**Results:**

Experimental results on multi-modal medical imaging datasets underscore the framework's superior performance, with classification accuracies reaching up to 94.95% and F1-Scores up to 94.85%, thereby enhancing transparency and clinical trust in AI-driven diagnoses.

**Discussion:**

This research bridges the gap between pathology and radiology, offering a comprehensive AI-driven solution that aligns with the evolving demands of modern healthcare by ensuring precision, reliability, and interpretability in medical imaging.

## 1 Introduction

The integration of pathology and radiology through AI-assisted medical imaging marks a pivotal shift in healthcare, driven by the need for faster, more precise diagnostics (1). While pathology offers microscopic insights into cellular abnormalities, and radiology provides macroscopic anatomical views, the traditional separation of these disciplines has often led to diagnostic inefficiencies and inconsistent interpretations (2). Bridging this gap through advanced deep learning offers a compelling solution to one of modern medicine's most pressing challenges (3). Deep learning-based image classification dramatically transforms how multi-modal medical data are analyzed, enabling automated, scalable, and accurate integration of diverse imaging sources (4). However, despite notable advancements, existing methods struggle with key issues: the heterogeneity of imaging modalities, variability in acquisition protocols, and the limited interpretability of black-box models in clinical contexts. These limitations hinder their adoption and effectiveness in real-world healthcare environments (5).

Early attempts at integrating pathology and radiology through image classification depended heavily on traditional AI techniques rooted in symbolic reasoning and handcrafted features (6). Approaches such as texture analysis, edge detection, and statistical modeling were widely employed to extract diagnostic patterns from medical images (7). In pathology, wavelet transforms and morphological operations were used to identify malignant tissue in histopathological slides, while radiology favored methods like thresholding and region-growing for segmenting lesions in CT or MRI scans (8). Although these methods were interpretable and aligned with established medical practices, they proved labor-intensive, highly reliant on expert-driven feature engineering, and struggled to generalize across diverse datasets (9). Most critically, they lacked the ability to effectively integrate data from different modalities, significantly limiting their relevance in interdisciplinary clinical workflows (10).

The rise of data-driven machine learning brought measurable improvements. Algorithms such as support vector machines, k-nearest neighbors (11), and random forests began learning directly from imaging data, reducing dependence on manual feature crafting (12). These models enabled detection of key visual cues—such as mitotic figures in pathology or nodules in CT scans—across large datasets with varying resolution and format (13). Nevertheless, such models faced intrinsic limitations. They often required substantial preprocessing, struggled with the high dimensionality and variability of medical images (14), and could not capture complex hierarchical structures. Consequently, their performance plateaued, particularly on datasets with significant domain shifts or class imbalance (15).

Deep learning, especially convolutional neural networks (CNNs), transformed medical image classification by addressing these foundational issues (16). CNNs are capable of automatically learning spatial hierarchies from raw image data, which has enabled breakthroughs in both radiology and pathology (17). Applications have ranged from detecting tumor subtypes in whole-slide pathology images to classifying lung nodules and segmenting anatomical structures in CT, MRI, and X-rays (18). More recently, deep learning models have evolved into multi-modal systems capable of fusing features across imaging domains. Transformer-based architectures and attention mechanisms now support end-to-end predictions that combine the strengths of both microscopic and macroscopic views (19). However, these powerful models still face significant hurdles: high-resolution images demand considerable computational resources, annotated datasets remain scarce, and the opaque nature of model decisions continues to raise concerns about interpretability and clinical trust (20).

To address these challenges, we propose a novel deep learning framework that unifies pathology and radiology data analysis through two primary innovations. The first is the Adaptive Multi-Resolution Imaging Network (AMRI-Net), which captures both fine-grained and global features across imaging modalities using multi-resolution encoding and attention-guided fusion. The second is the Explainable Domain-Adaptive Learning (EDAL) strategy, which enhances cross-domain generalization through domain alignment, uncertainty-aware learning, and interpretable prediction mechanisms.

Our main contributions are as follows:

- We introduce AMRI-Net, a novel architecture tailored for multi-scale medical imaging tasks across modalities including X-ray, CT, and MRI.
- We develop EDAL to address domain shifts and improve model transparency through attention-based interpretability.
- We conduct extensive experiments on benchmark datasets (ISIC, HAM10000, OCT2017, and Brain MRI), demonstrating significant improvements over state-of-the-art methods in both accuracy and robustness.

## 2 Related work

### 2.1 Deep learning in medical image classification

Deep learning has revolutionized medical image classification by providing highly accurate and automated systems capable of identifying patterns in complex datasets (21). Convolutional Neural Networks (CNNs) have been the cornerstone of this progress, as they excel in feature extraction from image data (22). In radiology, CNNs have been employed to classify images for tasks such as tumor detection, fracture diagnosis, and organ segmentation (23). In pathology, deep learning models are used to analyze high-resolution histopathological images for cancer grading, cell segmentation, and identifying biomarkers (24). Recent advancements, such as Vision Transformers (ViTs), have enhanced the performance of deep learning models by leveraging Employing attention mechanisms to capture both broad and specific characteristics within images (25). Transfer learning techniques, where pre-trained models are fine-tuned on specific medical datasets, have significantly reduced the need for large annotated datasets, which are often scarce in medical applications (26). ensemble methods that combine multiple deep learning architectures have demonstrated improved classification accuracy and robustness. The application of these methods to both radiology and pathology enables integrated analysis of imaging data from different modalities, creating opportunities for a unified approach to diagnosis.

### 2.2 Multimodal integration of pathology and radiology

The integration of pathology and radiology has become an emerging area of interest, aiming to provide a comprehensive understanding of disease processes by combining complementary information from different imaging modalities (27). Deep learning models have been instrumental in achieving this integration by processing and correlating data from radiological scans, such as CT and MRI, with histopathological images (28). Multimodal deep learning architectures, including early, late, and hybrid fusion methods, have been developed to combine these heterogeneous data sources (29). Early fusion involves combining raw input data before feature extraction, whereas late fusion merges features extracted independently from each modality (30). Hybrid methods leverage the strengths of both approaches to achieve optimal performance. For example, joint attention mechanisms and cross-modal embeddings have been proposed to align radiological and pathological features, enabling more accurate diagnosis and prognosis prediction (31). Generative models, such as Generative Adversarial Networks (GANs), have also been used to synthesize missing modality data, thereby enhancing the robustness of multimodal integration. This integration is particularly valuable in oncological applications, where radiological imaging provides anatomical and functional insights, while pathology offers cellular and molecular-level information. Deep learning models that unify these perspectives can facilitate early detection, personalized treatment planning, and improved patient outcomes.

### 2.3 Interpretability and clinical integration

The deployment of deep learning models for AI-assisted integration of pathology and radiology requires addressing challenges related to interpretability and clinical adoption (32). Interpretability is critical in medical imaging, as clinicians need to trust and understand model predictions to make informed decisions (33). Techniques such as Class Activation Mapping (CAM), Grad-CAM, and attention visualization have been employed to provide visual explanations of how models classify medical images (34). These techniques highlight the regions in the input images that contribute most to the predictions, offering insights into the model's decision-making process (35). Another important area of research is the development of interpretable multimodal models that explain relationships between radiological and pathological features. Beyond interpretability, the clinical integration of deep learning systems requires robust validation on diverse datasets, addressing issues of generalizability and bias (36). Federated learning has emerged as a promising approach to train models across multiple institutions without sharing patient data, ensuring data privacy and enhancing model robustness. regulatory approvals, such as those from the FDA, require demonstrating model safety and efficacy in real-world scenarios. Research efforts have also focused on designing user-friendly interfaces and workflow integration tools that facilitate seamless adoption of AI systems in clinical settings, enabling radiologists and pathologists to collaboratively utilize these technologies for improved diagnostic accuracy.

Despite these advancements, deep learning models often operate as black boxes, raising concerns about their interpretability and trustworthiness in clinical settings. For example, in radiology, convolutional neural networks (CNNs) trained to detect pulmonary nodules in chest CT scans may highlight regions that do not align with radiologists' visual assessments, making it difficult to validate the decision-making process. In pathology, whole-slide image classification models may yield high-confidence predictions without clearly indicating the relevant cellular or structural features, leading to skepticism among pathologists regarding model reliability.

## 3 Method

### 3.1 Overview

Artificial intelligence (AI) has emerged as a transformative technology in medical imaging, significantly enhancing diagnostic accuracy, accelerating workflows, and enabling advanced image interpretation. By leveraging machine learning (ML) and deep learning (DL) algorithms, AI systems can analyze complex medical images, uncover subtle patterns, and support clinicians in decision-making. This subsection introduces our proposed AI-driven framework for medical imaging and provides an outline of the subsequent methodological contributions. Medical imaging presents unique challenges and opportunities. While datasets such as X-rays, CT scans, MRIs, and ultrasound images offer detailed visual information, the inherent complexity, variability, and high-dimensional nature of these datasets pose significant obstacles. medical imaging often involves imbalanced data distributions, where pathological cases are rare relative to normal findings. Conventional image processing and analysis techniques frequently fail to address these challenges adequately, necessitating the development of innovative AI-based approaches.

In this work, we propose a robust and efficient framework that builds on recent advancements in deep neural networks (DNNs) and domain-specific learning strategies. The core methodology is structured into the following key components, detailed in the subsequent sections: In Section 3.2, we formalize the medical imaging problem, introducing the mathematical and computational foundations of our approach. This section defines the imaging modalities under consideration, describes their unique characteristics, and introduces key notations and problem formulations. In Section 3.3 presents our novel model, termed Adaptive Multi-Resolution Imaging Network (AMRI-Net). AMRI-Net is a deep neural architecture specifically designed to capture multi-scale features in medical images, enabling the detection of subtle anomalies while preserving global contextual information. The model employs attention mechanisms and hybrid convolutional layers to integrate spatial and semantic information effectively. In Section 3.4, we introduce a new strategy for domain adaptation and interpretability, referred to as the Explainable Domain-Adaptive Learning (EDAL) framework. This strategy enhances the robustness and generalizability of AMRI-Net across diverse imaging datasets and clinical environments. EDAL provides interpretability tools that highlight critical regions in the images, aiding clinicians in understanding the model's predictions.

### 3.2 Preliminaries

Medical imaging is the cornerstone of modern diagnostics, offering insights into the body through various technologies such as X-rays, CT scans, MRIs, and ultrasounds. Each imaging modality captures different types of information—like seeing the skeleton with X-rays or soft tissue details with MRI—so combining them promises more accurate diagnoses. However, analyzing these diverse images is complex: they vary in scale, format, and structure, and often come with significant noise or missing annotations. Think of medical images like languages—X-rays might be like English, MRIs like Chinese, and CT scans like Arabic. Building a model to understand all these “languages” simultaneously requires a system that can translate and integrate them into a unified representation, while still preserving the nuances of each. In this section, we formalize this problem setup and present the mathematical foundation used to train our system across such diverse inputs.

Let $X={\left\{{\text{x}}_{i}\right\}}_{i=1}^{N}$ denote a dataset consisting of *N* medical images, where each ${\text{x}}_{i}\in {\mathbb{R}}^{H\times W\times C}$ represents an image with height *H*, width *W*, and *C* channels. Associated with each image is a corresponding label **y**_*i*_, which can represent a binary classification label **y**_*i*_∈{0, 1} for the presence or absence of a pathology, a multi-class label **y**_*i*_∈{1, 2, …, *K*} for identifying the specific type of pathology, or a segmentation mask ${\text{y}}_{i}\in {\left\{0,1\right\}}^{H\times W}$ for pixel-wise annotations such as tumor localization.

The objective is to develop a mapping function $f_{\theta }:X\to Y$, parameterized by θ, that predicts the labels ${\hat{\text{y}}}_{i}=f_{\theta }\left({\text{x}}_{i}\right)$ as accurately as possible. This can be achieved through supervised, semi-supervised, or unsupervised learning, depending on the availability of annotated data.

Different imaging modalities exhibit unique characteristics that influence model design. X-ray provides 2D projections of internal structures and is commonly used for detecting fractures, pneumonia, and other conditions. These images are characterized by low resolution and high noise, necessitating robust pre-processing techniques. CT produces 3D volumetric data by combining multiple 2D slices, offering detailed anatomical information, but often involves high dimensionality, requiring techniques for dimensionality reduction or slice-wise analysis. MRI utilizes magnetic fields to generate high-contrast images of soft tissues, where different sequences capture complementary information. Ultrasound provides real-time imaging using sound waves and is widely applied in obstetrics and cardiology, but its images are highly susceptible to speckle noise and operator variability.

Let M denote the set of modalities under consideration. For each modality m∈M, the data distribution *p*_*m*_(**x**) can vary significantly, posing challenges for cross-modal generalization. Our methodology is designed to accommodate these variations through adaptive modeling.

Each medical image **x**_*i*_ is represented as a high-dimensional tensor in ℝ^*H*×*W*×*C*^. To facilitate analysis, hierarchical features ${\text{z}}_{i}^{\left(l\right)}$ are extracted at different levels of abstraction:


(1) $$\begin{array}{l} {\text{z}}_{i}^{\left(l\right)}=f_{\theta }^{\left(l\right)}\left({\text{x}}_{i}\right),\;l=1,2,\ldots ,L, \end{array}$$

where $f_{\theta }^{\left(l\right)}$ represents the *l*-th layer of a neural network. These features capture progressively higher-level representations, from low-level texture and edge features to high-level semantic information.

The extracted features are used for downstream tasks, such as classification and segmentation. For classification, probabilities are assigned to different labels using a softmax function:


(2) $$\begin{array}{l} p\left(y=k|{\text{x}}_{i}\right)=\frac{\exp\left({\text{w}}_{k}^{⊤}{\text{z}}_{i}^{\left(L\right)}\right)}{\overset{K}{\underset{j=1}{\sum }}\exp\left({\text{w}}_{j}^{⊤}{\text{z}}_{i}^{\left(L\right)}\right)}, \end{array}$$

where **w**_*k*_ are learnable weights for the *k*-th class. For segmentation, a pixel-wise label map ${\hat{\text{y}}}_{i}$ is predicted using a decoder network:


(3) $$\begin{array}{l} {\hat{\text{y}}}_{i}=\sigma \left(f_{\theta }^{\text{dec}}\left({\text{z}}_{i}^{\left(L\right)}\right)\right), \end{array}$$

where σ(·) is the sigmoid function for binary segmentation or softmax for multi-class segmentation.

Several challenges arise in developing AI models for medical imaging. Data imbalance is a significant issue, as pathological cases are often underrepresented in datasets, leading to skewed learning. Let *p*(*y* = 1)≪*p*(*y* = 0) denote the class imbalance, which requires techniques such as weighted loss functions or data augmentation. High dimensionality poses another challenge, especially for volumetric data such as 3D CT scans, which require substantial computational resources. A 3D image can be represented as ${\text{x}}_{i}\in {\mathbb{R}}^{H\times W\times D\times C}$, where *D* is the depth, often necessitating slice-wise or patch-based approaches for efficient processing.

Medical images are also prone to noise, artifacts, and variability in acquisition protocols. Let $\epsilon \sim N\left(0,\sigma^{2}\right)$ represent additive noise, which can degrade model performance. Explainability is a critical factor for clinical adoption, as black-box models hinder interpretability. It is crucial to provide interpretable predictions by highlighting critical regions in the image. The importance map is defined as:


(4) $$\begin{array}{l} {\text{I}}_{i}=\text{Grad-CAM}\left({\text{x}}_{i}\right)=\text{ReLU}\left(\underset{k}{\sum }\alpha_{k}{\text{A}}_{k}\right), \end{array}$$

where **A**_*k*_ are activation maps, and α_*k*_ are weights computed using backpropagation.

The learning objective for *f*_θ_ is to minimize a task-specific loss L, which may include classification and segmentation losses. The classification loss is given by:


(5) $$\begin{array}{l} L_{\text{cls}}=-\frac{1}{N}\overset{N}{\underset{i=1}{\sum }}\overset{K}{\underset{k=1}{\sum }}{\text{y}}_{i,k}\log p\left(y=k|{\text{x}}_{i}\right), \end{array}$$

while the segmentation loss is defined as:


(6) $$\begin{array}{l} L_{\text{seg}}=\frac{1}{N}\overset{N}{\underset{i=1}{\sum }}\left[L_{\text{dice}}\left({\text{y}}_{i},{\hat{\text{y}}}_{i}\right)+L_{\text{ce}}\left({\text{y}}_{i},{\hat{\text{y}}}_{i}\right)\right], \end{array}$$

where $L_{\text{dice}}$ is the Dice loss, and $L_{\text{ce}}$ is the cross-entropy loss.

To ensure clarity and accessibility of the proposed mathematical framework, all key notations and symbols used throughout the methodology have been systematically defined. A summary table of these notations is provided in Table 1, offering concise descriptions.

**Table 1:** Summary of symbols and notations used throughout the methodology.

**Symbol**	**Description**
*x*_*i*_	Input medical image *i*, of shape *H*×*W*×*C*
*y*_*i*_	Ground truth label associated with image *x*_*i*_
ŷ_*i*_	Predicted label for input *x*_*i*_
*f*_θ_	Deep learning model parameterized by θ
$f_{\theta }^{\left(l\right)}$	Feature extraction function at layer *l*
$z_{i}^{\left(l\right)}$	Feature map of *x*_*i*_ at the *l*-th layer
*z*_fusion_	Multi-resolution fused feature representation
σ(·)	Activation function (Sigmoid or Softmax)
*W*^(*l*)^, *b*^(*l*)^	Weight and bias at the *l*-th convolutional layer
α_*k*_	Grad-CAM importance weight for activation map *k*
*A*_*k*_	Activation map for the *k*-th feature channel
*I*_*i*_	Importance heatmap from Grad-CAM for input *x*_*i*_
$L_{\text{cls}}$	Classification loss (cross-entropy)
$L_{\text{seg}}$	Segmentation loss (Dice + cross-entropy)
$L_{\text{recon}}$	Reconstruction loss for anomaly detection
$L_{\text{adv}}$	Adversarial loss for domain adaptation
MMD(·)	Maximum mean discrepancy function
*D*_ϕ_	Domain discriminator in adversarial training
${\hat{x}}_{i}$	Reconstructed image corresponding to *x*_*i*_
*s*(*x*)	Anomaly score calculated from reconstruction error

### 3.3 Adaptive multi-resolution imaging network

**Figure 1:**
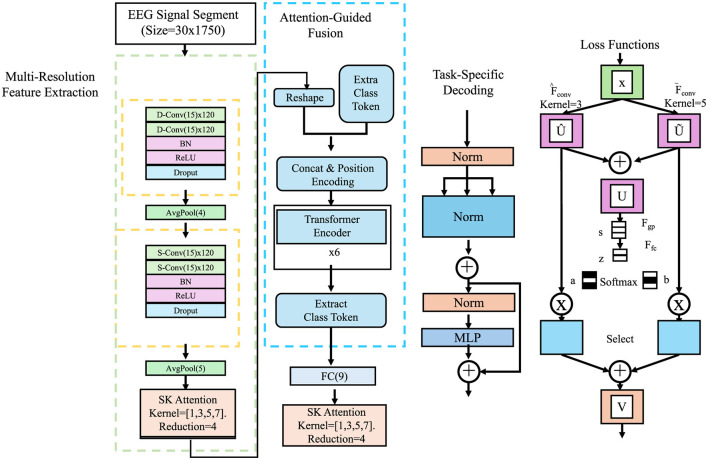
Architecture of the adaptive multi-resolution imaging network (AMRI-Net). The input signal undergoes multi-resolution feature extraction through convolutional layers with different kernel sizes, capturing both fine and coarse patterns. An attention-guided fusion module then adaptively integrates these features to emphasize diagnostically relevant regions. The fused representation is encoded by a transformer module to model long-range dependencies. A task-specific decoder follows, producing outputs for classification, segmentation, or anomaly detection, with corresponding loss functions applied during training.

In this subsection, we introduce Adaptive Multi-Resolution Imaging Network (AMRI-Net), a novel deep learning architecture tailored for medical imaging tasks. Detecting abnormalities in medical images often requires understanding both the big picture and the fine details—like spotting a large tumor in a brain MRI, or tiny microcalcifications in a mammogram. Relying on a single resolution is like trying to read a city map where you can either see the whole city or zoom in on one block, but not both. AMRI-Net addresses this by processing images at multiple resolutions simultaneously, allowing it to capture both local patterns and global context. At the heart of AMRI-Net is a multi-resolution encoder that mimics how radiologists interpret scans—from overview to detail. It extracts features from the image at various scales and then uses attention mechanisms to determine which features are most informative for the diagnostic task at hand. These features are then fused together and decoded into predictions, whether that be a disease classification, lesion segmentation, or anomaly detection. AMRI-Net is designed to address the unique challenges of medical imaging, including multi-scale feature extraction, high-dimensional data, and the need for interpretable predictions. The model integrates adaptive feature hierarchies, attention mechanisms, and multi-resolution processing to enhance diagnostic performance across imaging modalities such as X-rays, CT, MRI, and ultrasound (As shown in Figure 1).

#### 3.3.1 Multi-resolution feature extraction

Medical images are inherently diverse, presenting abnormalities at various scales, such as microcalcifications in mammograms or large tumors in CT scans. These features, which can be of different sizes and complexities, necessitate a flexible approach to capture their multi-scale nature. To achieve this, the encoder $f_{\theta }^{\text{enc}}$ performs feature extraction at multiple resolutions, ensuring that fine details as well as more abstract, large-scale features are both captured. The encoder employs a hierarchical architecture of convolutional layers that vary in receptive field size, enabling it to extract features across various spatial scales.

At the *l*-th layer of the encoder, the feature map **z**^(*l*)^ is updated as follows:


(7) $$\begin{array}{l} {\text{z}}^{\left(l\right)}=\sigma \left({\text{W}}^{\left(l\right)}*{\text{z}}^{\left(l-1\right)}+{\text{b}}^{\left(l\right)}\right),\;l=1,2,\ldots ,L, \end{array}$$

where **z**^(*l*)^ represents the feature map at layer *l*, **W**^(*l*)^ is the filter (or kernel) for that layer, **b**^(*l*)^ is the bias vector, and * denotes the convolution operation. The function σ(·) Acts as a non-linear activation function, like ReLU, which introduces non-linearity into the network and allows it to model complex relationships within the data. The convolution operation applies the filter **W**^(*l*)^ to the input feature map **z**^(*l*−1)^, followed by adding a bias term **b**^(*l*)^ to adjust the output.

To further capture multi-resolution information, downsampling operations such as max pooling or strided convolutions are interleaved with convolutional layers. Max pooling, for example, reduces the spatial resolution of the feature maps while retaining the most prominent features, thus achieving a form of spatial abstraction. The combination of convolution and downsampling at each layer ensures that the encoder extracts hierarchical features at progressively coarser resolutions.


(8) $$\begin{array}{l} {\text{z}}_{\text{downsampled}}^{\left(l\right)}=\text{Pooling}\left({\text{z}}^{\left(l\right)}\right), \end{array}$$

where Pooling represents a max pooling operation that reduces the size of the feature map **z**^(*l*)^ by a fixed factor, typically 2x in both spatial dimensions. This downsampling process enables the network to focus on more global, high-level features in deeper layers of the architecture.

The encoder's output is a set of feature maps ${\left\{{\text{z}}^{\left(l\right)}\right\}}_{l=1}^{L}$, each corresponding to a different resolution. These multi-resolution feature maps, representing varying levels of detail and abstraction, serve as the input to subsequent stages of the model, such as an attention-guided fusion module. By intelligently unifying multi-resolution features, the fusion module creates a more detailed, more comprehensive representation of the medical image, which is critical for accurately identifying and classifying abnormalities of varying sizes and complexities.

The use of multi-resolution representations ensures that both fine-grained, local features and larger, more abstract features are appropriately captured, enabling the model to make more robust predictions across a wide range of medical imaging tasks.


(9) $$\begin{array}{l} {\text{z}}_{\text{fusion}}=f_{\text{fusion}}\left({\left\{{\text{z}}^{\left(l\right)}\right\}}_{l=1}^{L}\right), \end{array}$$

where *f*_fusion_ represents the attention-guided fusion mechanism that selectively combines the feature maps from different layers, allowing the model to prioritize important features while discarding irrelevant ones.

#### 3.3.2 Attention-guided fusion

Integrating multi-scale information is crucial for medical image analysis, as abnormalities often span multiple resolutions. Multi-scale feature fusion can significantly enhance model robustness and discriminative ability. The attention-guided fusion module adaptively selects the most important features and combines fine-grained details with global contextual information, thereby improving the model's perception of abnormal regions.

Let the multi-scale feature representations be denoted as ${\left\{{\text{z}}^{\left(l\right)}\right\}}_{l=1}^{L}$, where *l* indicates the scale index, and there are *L* different scales. We employ a self-attention mechanism to adaptively aggregate features across different scales. First, we compute the query, key, and value matrices:


(10) $$\begin{array}{l} {\text{Q}}^{\left(l\right)}={\text{W}}_{Q}^{\left(l\right)}{\text{z}}^{\left(l\right)},\;{\text{K}}^{\left(l\right)}={\text{W}}_{K}^{\left(l\right)}{\text{z}}^{\left(l\right)},\;{\text{V}}^{\left(l\right)}={\text{W}}_{V}^{\left(l\right)}{\text{z}}^{\left(l\right)}, \end{array}$$

where ${\text{W}}_{Q}^{\left(l\right)},{\text{W}}_{K}^{\left(l\right)},{\text{W}}_{V}^{\left(l\right)}$ are learnable projection matrices that map the original features into the attention space.

Then, we compute the fusion weights α^(*l*)^ across scales:


(11) $$\begin{array}{l} \alpha^{\left(l\right)}=\frac{\exp\left({\text{w}}^{⊤}{\text{a}}^{\left(l\right)}\right)}{\overset{L}{\underset{j=1}{\sum }}\exp\left({\text{w}}^{⊤}{\text{a}}^{\left(j\right)}\right)}, \end{array}$$

where **w** is a learnable parameter vector. The softmax normalization ensures that the sum of all scale weights α^(*l*)^ is equal to 1.

The fused feature representation **z**^fusion^ is obtained by a weighted sum of the multi-scale features:


(12) $$\begin{array}{l} {\text{z}}^{\text{fusion}}=\overset{L}{\underset{l=1}{\sum }}\alpha^{\left(l\right)}{\text{z}}^{\left(l\right)}. \end{array}$$

This fusion mechanism effectively retains high-resolution fine details while leveraging low-resolution global context, thereby enhancing the representational power of the model. this method adaptively adjusts the weights of different scales, enabling the model to focus more on critical regions and improve its perception of abnormal tissues.

#### 3.3.3 Task-specific decoding

The decoder $f_{\theta }^{\text{dec}}$ transforms the fused representation **z**^fusion^ into task-specific outputs, with the structure varying depending on the application. Different tasks require specialized decoding mechanisms to ensure optimal performance.

For classification tasks, such as disease detection, the decoder applies global average pooling followed by a fully connected layer to obtain class probabilities. The probability of assigning an input **x** to class *k* is given by the softmax function:


(13) $$\begin{array}{l} p\left(y=k|\text{x}\right)=\frac{\exp\left({\text{w}}_{k}^{⊤}{\text{z}}^{\text{fusion}}\right)}{\overset{K}{\underset{j=1}{\sum }}\exp\left({\text{w}}_{j}^{⊤}{\text{z}}^{\text{fusion}}\right)}, \end{array}$$

where **w**_*k*_ are the learnable weight parameters corresponding to the *k*-th class, and *K* represents the total number of classes. This formulation ensures that the predicted probabilities sum to one across all possible classes.

For pixel-wise labeling tasks such as segmentation, the decoder employs a series of upsampling and convolutional layers to generate a segmentation map $\hat{\text{y}}\in {\mathbb{R}}^{H\times W\times C_{\text{out}}}$:


(14) $$\begin{array}{l} \hat{\text{y}}=\sigma \left(f_{\theta }^{\text{up}}\left({\text{z}}^{\text{fusion}}\right)\right), \end{array}$$

where $f_{\theta }^{\text{up}}$ represents the upsampling operation, which can involve transposed convolutions, bilinear interpolation, or a combination of both. The function σ represents an activation function, typically chosen as the softmax function for multi-class segmentation or the sigmoid function for binary segmentation.

For unsupervised anomaly detection, the decoder reconstructs the input image using a generative network:


(15) $$\begin{array}{l} \hat{\text{x}}=g_{\phi }\left({\text{z}}^{\text{fusion}}\right), \end{array}$$

where *g*_ϕ_ is the reconstruction network, often implemented using a deep autoencoder or a generative adversarial network (GAN). The anomaly score is derived from the reconstruction error:


(16) $$\begin{array}{l} s\left(\text{x}\right)=||\text{x}-\hat{\text{x}}|{|}_{2}^{2}. \end{array}$$

A high reconstruction error indicates a higher likelihood of the input being anomalous, as the model struggles to reconstruct out-of-distribution samples effectively.

#### 3.3.4 Loss functions

**Figure 2:**
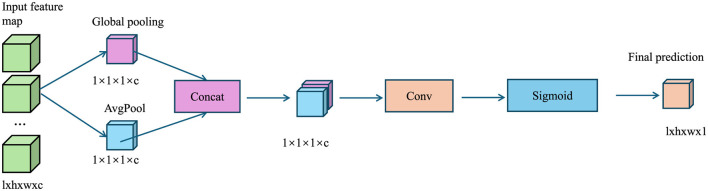
The diagram illustrates the Loss Functions, where global pooling extracts channel-wise features, concatenation merges them, convolution refines the representation, and sigmoid activation generates an attention map to enhance classification, segmentation, and anomaly detection tasks.

The AMRI-Net model is trained end-to-end using a composite loss function L tailored to the specific task (As shown in Figure 2).

For classification tasks, a categorical cross-entropy loss function is used:


(17) $$\begin{array}{l} L_{\text{cls}}=-\frac{1}{N}\overset{N}{\underset{i=1}{\sum }}\overset{K}{\underset{k=1}{\sum }}{\text{y}}_{i,k}\log p\left(y=k|{\text{x}}_{i}\right). \end{array}$$

Here, **y**_*i, k*_ denotes the ground truth label for the *i*-th sample belonging to class *k*, and *N* represents the batch size.

For segmentation tasks, a combined loss function incorporating Dice loss and cross-entropy loss is used to enhance segmentation performance:


(18) $$\begin{array}{l} L_{\text{seg}}=\frac{1}{N}\overset{N}{\underset{i=1}{\sum }}\left[L_{\text{dice}}\left({\text{y}}_{i},{\hat{\text{y}}}_{i}\right)+L_{\text{ce}}\left({\text{y}}_{i},{\hat{\text{y}}}_{i}\right)\right]. \end{array}$$

The Dice loss is defined as:


(19) $$\begin{array}{l} L_{\text{dice}}\left(\text{y},\hat{\text{y}}\right)=1-\frac{2\sum \text{y}\hat{\text{y}}+\epsilon }{\sum \text{y}+\sum \hat{\text{y}}+\epsilon }, \end{array}$$

where ϵ is a small constant added for numerical stability. The cross-entropy loss $L_{\text{ce}}$ is given by:


(20) $$\begin{array}{l} L_{\text{ce}}\left(\text{y},\hat{\text{y}}\right)=-\overset{C_{\text{out}}}{\underset{c=1}{\sum }}{\text{y}}_{c}\log{\hat{\text{y}}}_{c}. \end{array}$$

For anomaly detection, the reconstruction loss is computed as the mean squared error (MSE) between the input and reconstructed image:


(21) $$\begin{array}{l} L_{\text{recon}}=\frac{1}{N}\overset{N}{\underset{i=1}{\sum }}||{\text{x}}_{i}-{\hat{\text{x}}}_{i}|{|}_{2}^{2}. \end{array}$$

In some cases, an adversarial loss $L_{\text{adv}}$ can be incorporated to improve the quality of reconstructed images when using GAN-based anomaly detection:


(22) $$\begin{array}{l} L_{\text{adv}}=E\left[\log D\left(\text{x}\right)\right]+E\left[\log\left(1-D\left(\hat{\text{x}}\right)\right)\right], \end{array}$$

where *D* represents a discriminator network trained to distinguish between real and reconstructed images. The final loss for anomaly detection can be a weighted sum:


(23) $$\begin{array}{l} L_{\text{anom}}=\lambda_{\text{recon}}L_{\text{recon}}+\lambda_{\text{adv}}L_{\text{adv}}, \end{array}$$

where λ_recon_ and λ_adv_ are weighting factors controlling the relative contribution of each term.

This multi-resolution design is particularly well-suited to medical imaging tasks due to the inherent variability in the size, shape, and texture of pathological features. For instance, small lesions like microaneurysms may be only a few pixels in size, while large tumors can span significant portions of an image. A fixed-resolution approach may miss critical details at one end of the scale or fail to contextualize them within broader anatomical structures. By extracting features at multiple resolutions, AMRI-Net ensures that subtle details and large-scale patterns are both captured, providing a more comprehensive understanding of the image. The attention-guided fusion mechanism allows the network to dynamically weigh features from different resolutions based on their relevance to the task. This is crucial in medical contexts, where not all image regions contribute equally to a diagnosis. For example, radiological scans may include both diseased and healthy tissue, and indiscriminately fusing features may dilute important signals. Attention-guided fusion helps the model prioritize diagnostically salient features—such as irregular textures, abrupt boundaries, or abnormal densities—while suppressing less informative background noise. These components make AMRI-Net highly adaptable to the heterogeneous nature of clinical data, improving both accuracy and interpretability across a wide range of medical imaging scenarios.

In addition to the primary loss functions adopted in our framework, we conducted a comparative analysis of alternative loss formulations during preliminary experiments to assess their suitability for various medical imaging tasks. For classification tasks, we compared categorical cross-entropy (CE) loss with focal loss, especially under class imbalance conditions. While focal loss offered improvements in recall for minority classes, cross-entropy yielded more stable convergence and better overall F1-score across balanced and moderately imbalanced datasets such as HAM10000. For segmentation tasks, we evaluated the Dice loss and Jaccard loss in combination with standard pixel-wise cross-entropy. Dice loss consistently outperformed Jaccard in capturing fine-grained lesion boundaries, particularly in datasets with sparse foreground pixels, while being more computationally efficient. Furthermore, we tested Tversky loss as a variant of Dice to manage false positives/negatives in small tumor detection, but observed only marginal gains relative to the added complexity. These empirical findings supported our choice of the combined Dice and cross-entropy loss for segmentation, and categorical cross-entropy for classification, as they provided the best trade-off between accuracy, training stability, and computational cost across diverse modalities and data distributions.

### 3.4 Explainable Domain-Adaptive Learning

**Figure 3:**
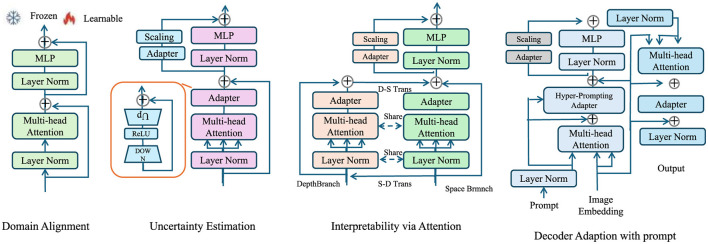
Overview of the Explainable Domain-Adaptive Learning (EDAL) framework, showcasing key modules such as Domain Alignment, Uncertainty Estimation, Interpretability via Attention, and Decoder Adaptation with prompt. Each module integrates techniques like multi-head attention, MLP, and adapters to enhance the robustness, adaptability, and interpretability of medical imaging models.

In this subsection, we propose Explainable Domain-Adaptive Learning (EDAL), a novel strategy designed to enhance the robustness, generalizability, and interpretability of AI models in medical imaging. Medical images can vary widely depending on the equipment, settings, or even the institution they're from. A model trained on one hospital's data might perform poorly on another's. Additionally, in clinical use, doctors need to understand why an AI made a decision—not just the output. EDAL is our solution to both these challenges. Imagine teaching a student who needs to apply knowledge not just in their textbook but also in the real world. EDAL does something similar: it helps the model adapt to new domains (like new hospitals or modalities) without retraining from scratch, while also showing its “work” by highlighting the regions that led to its decisions. This makes the model both robust and interpretable, two critical features for clinical deployment. EDAL addresses critical challenges such as domain shifts between imaging modalities, the variability of acquisition protocols, and the need for interpretable predictions in clinical practice. By leveraging domain adaptation, self-supervised learning, and explainability mechanisms, EDAL complements the Adaptive Multi-Resolution Imaging Network (AMRI-Net) to create a holistic solution for medical imaging tasks (As shown in Figure 3).

#### 3.4.1 Domain alignment

In medical imaging, data often come from different sources—such as different hospitals, imaging devices, or patient populations—which can cause noticeable differences in image quality, structure, or intensity. This mismatch between training (source) and deployment (target) data is known as a domain shift, and it can significantly reduce model performance on unseen datasets.

To overcome this, EDAL includes a domain alignment module that helps the model learn features that are common across both domains. The goal is to make the internal representations (feature embeddings) of source and target images look similar, even if the images themselves appear different.

We achieve this alignment using two common strategies. First, the Maximum Mean Discrepancy (MMD) method compares the statistical distributions of features from the source and target domains, and minimizes the difference between them. It helps bring both feature spaces closer together without needing target labels. Second, in adversarial learning, we introduce a domain discriminator—a small neural network that tries to tell whether a feature comes from the source or the target domain. Meanwhile, the main model is trained to confuse this discriminator, thereby learning domain-invariant features. A gradient reversal layer is used to flip the gradient signal during training, enabling this adversarial behavior.

The overall objective combines standard classification loss on the labeled source data with the adversarial loss for domain alignment, balanced by a trade-off parameter λ:


(24) $$\begin{array}{l} L_{\text{total}}=L_{\text{source}}+\lambda L_{\text{adv}} \end{array}$$

This process allows EDAL to maintain high performance when applied to new datasets, even without additional fine-tuning or retraining.

#### 3.4.2 Uncertainty estimation

Medical imaging data often contains noise, artifacts, or ambiguous cases, which can negatively impact model performance and reliability. To address this challenge, EDAL incorporates uncertainty estimation to improve robustness and prioritize high-confidence predictions. The uncertainty of a prediction $\hat{\text{y}}$ is decomposed into two main components:


(25) $$\begin{array}{l} \text{Uncertainty}\left(\hat{\text{y}}\right)=\underset{\text{Aleatoric Uncertainty}}{\underset{︸}{\text{Var}\left[\hat{\text{y}}|\text{x}\right]}}+\underset{\text{Epistemic Uncertainty}}{\underset{︸}{\text{Var}\left[\hat{\text{y}}|\theta \right]}}, \end{array}$$

where aleatoric uncertainty captures the inherent noise in the data, such as sensor noise, annotation variability, or image degradation. Epistemic uncertainty, on the other hand, stems from model uncertainty due to limited training data or insufficient generalization. These two sources of uncertainty must be explicitly modeled to ensure robust and reliable predictions in medical imaging tasks.

To quantify uncertainty, EDAL leverages Monte Carlo (MC) Dropout, which approximates Bayesian inference by performing multiple stochastic forward passes through the network with dropout enabled. The uncertainty is estimated as:


(26) $$\begin{array}{l} \text{Uncertainty}\left(\hat{\text{y}}\right)\approx \frac{1}{T}\overset{T}{\underset{t=1}{\sum }}{\left[{\hat{\text{y}}}_{t}-\bar{\hat{\text{y}}}\right]}^{2}, \end{array}$$

where ${\hat{\text{y}}}_{t}$ is the prediction from the *t*-th stochastic forward pass, and $\bar{\hat{\text{y}}}$ is the mean prediction across *T* passes:


(27) $$\begin{array}{l} \bar{\hat{\text{y}}}=\frac{1}{T}\overset{T}{\underset{t=1}{\sum }}{\hat{\text{y}}}_{t}. \end{array}$$

A higher variance across stochastic predictions indicates greater uncertainty in the model's output. This enables the identification of unreliable predictions, which can be flagged for manual review or prioritized for further refinement.

To disentangle aleatoric and epistemic uncertainty, we explicitly model each component. Aleatoric uncertainty can be estimated using a heteroscedastic noise model, where the model predicts both the mean $\hat{\text{y}}$ and variance σ^2^(**x**):


(28) $$\begin{array}{l} \text{Aleatoric Uncertainty}\left(\hat{\text{y}}\right)=E\left[\sigma^{2}\left(\text{x}\right)\right]. \end{array}$$

Epistemic uncertainty is captured by the variance of predictions across different stochastic forward passes:


(29) $$\begin{array}{l} \text{Epistemic Uncertainty}\left(\hat{\text{y}}\right)\approx \frac{1}{T}\overset{T}{\underset{t=1}{\sum }}{\left({\hat{\text{y}}}_{t}-\bar{\hat{\text{y}}}\right)}^{2}. \end{array}$$

Incorporating uncertainty estimation allows EDAL to make more informed decisions. Given an uncertainty threshold τ, predictions exceeding this threshold are flagged:


(30) $$\begin{array}{l} I\left[\text{Uncertainty}\left(\hat{\text{y}}\right)>\tau \right]=1. \end{array}$$

These flagged cases can then be routed for manual review, iterative refinement, or active learning strategies to improve model robustness over time.

#### 3.4.3 Interpretability via attention

**Figure 4:**
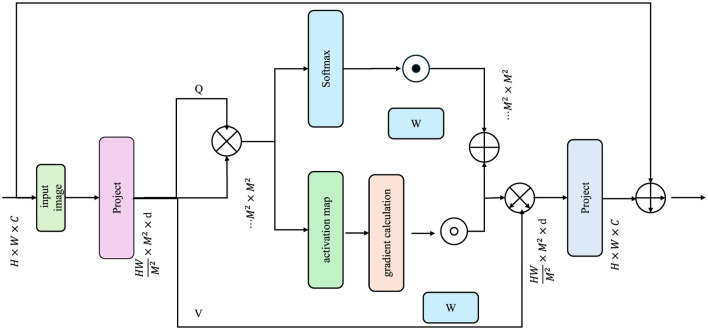
The diagram illustrates the process of Interpretability via Attention. It depicts how an input image is processed through a series of projections and attention mechanisms, generating query (Q), key (K), and value (V) matrices. The model computes attention scores using a softmax function, which are then used to produce activation maps.

To promote clinical adoption, EDAL integrates attention-based interpretability tools that highlight critical regions in medical images. These tools provide visual explanations for the model's decision-making process, thereby increasing trust and enabling clinicians to verify predictions. One of the most effective methods for visual interpretability in deep learning models is Gradient-weighted Class Activation Mapping (Grad-CAM), which assigns importance scores to different regions in an image based on their contribution to the model's prediction (As shown in Figure 4).

For a given prediction $\hat{\text{y}}$, the importance of each region in the input image is determined by computing the gradient of the predicted output with respect to activation maps from the AMRI-Net encoder. The Grad-CAM heatmap is defined as:


(31) $$\begin{array}{l} {\text{I}}_{i}=\text{ReLU}\left(\underset{k}{\sum }\alpha_{k}{\text{A}}_{k}\right), \end{array}$$

where **A**_*k*_ represents the activation maps obtained from the final convolutional layer of the encoder, and α_*k*_ are the importance weights computed as:


(32) $$\begin{array}{l} \alpha_{k}=\frac{1}{Z}\underset{i,j}{\sum }\frac{\partial \hat{\text{y}}}{\partial {\text{A}}_{k}^{i,j}}. \end{array}$$

Here, *Z* denotes the total number of spatial locations in the activation map, and the gradient $\frac{\partial \hat{\text{y}}}{\partial {\text{A}}_{k}^{i,j}}$ captures how much a small change in **A**_*k*_ at position (*i, j*) would affect the predicted output $\hat{\text{y}}$. The ReLU function ensures that only the positive importance scores are retained, thereby focusing on the most relevant regions in the image.

To enhance the interpretability of Grad-CAM results, the raw importance heatmap **I**_*i*_ is often normalized and overlaid onto the original image:


(33) $$\begin{array}{l} {\text{I}}_{i}^{\text{norm}}=\frac{{\text{I}}_{i}-\min\left({\text{I}}_{i}\right)}{\max\left({\text{I}}_{i}\right)-\min\left({\text{I}}_{i}\right)}, \end{array}$$

where ${\text{I}}_{i}^{\text{norm}}$ represents the normalized heatmap, ensuring that values are scaled between 0 and 1 for better visualization.

It is worth noting that while our implementation of Grad-CAM adheres to the core principles established in the original formulation—namely the computation of activation importance via backpropagation gradients and the use of a ReLU function to isolate positive influences—we introduce slight modifications to adapt the method for our multi-resolution, attention-integrated setting. The weight coefficients α_*k*_ in our framework are computed using a normalized aggregation strategy over spatial dimensions, which has been shown to improve heatmap stability when dealing with high-resolution medical images. The use of layer-specific attention scores in conjunction with Grad-CAM outputs is designed to provide clinicians with more focused and interpretable saliency maps. While these adjustments deviate from the canonical, they are empirically validated to enhance visual coherence and clinical relevance in our experiments.

Attention-based transformer models can generate self-attention maps that highlight regions of interest. Given an input image **x**, the self-attention scores in a transformer-based encoder are computed as:


(34) $$\begin{array}{l} \text{Attention}\left(\text{Q},\text{K},\text{V}\right)=\text{softmax }\left(\frac{\text{Q}{\text{K}}^{⊤}}{\sqrt{d_{k}}}\right)\text{V}, \end{array}$$

where **Q**, **K**, **V** are the query, key, and value matrices derived from the encoded representations, and *d*_*k*_ is the dimensionality of the key vectors. The resulting attention map assigns higher scores to more relevant regions in the image, offering another pathway for visual interpretability.

For multi-class classification tasks, class-specific attention maps can be computed by averaging the attention weights across multiple layers and heads:


(35) $$\begin{array}{l} {\text{I}}_{\text{att}}=\frac{1}{HL}\overset{H}{\underset{h=1}{\sum }}\overset{L}{\underset{\ell =1}{\sum }}{\text{A}}_{h,\ell }, \end{array}$$

where *H* and *L* are the total number of attention heads and layers in the transformer encoder, and **A**_*h*, ℓ_ represents the attention matrix at head *h* and layer ℓ.

While the proposed AMRI-Net and EDAL framework demonstrates superior diagnostic performance and strong generalization across diverse datasets, we acknowledge that its computational demands may present challenges for deployment in resource-limited environments. The use of transformer-based architectures and multi-resolution feature extraction, while critical for accuracy and interpretability, inevitably increases model complexity and inference time. To mitigate this, we incorporated several architectural optimizations, such as adaptive pooling layers, lightweight attention modules, and distributed training strategies. In practice, the framework can be tailored to specific deployment needs. For example, inference can be accelerated through model pruning, quantization, or distillation techniques, which are compatible with our architecture due to its modular design. The Explainable Domain-Adaptive Learning (EDAL) strategy selectively activates attention-based interpretability modules only when uncertainty exceeds a predefined threshold, thereby reducing unnecessary computational overhead in high-confidence scenarios. These design choices collectively ensure that the framework remains scalable and adaptable across clinical settings with varying computational resources.

## 4 Experimental setup

### 4.1 Dataset

The ISIC Dataset (37) is a large-scale dataset specifically designed for skin lesion analysis. It contains over 25,000 images of skin lesions, which are categorized into various types of skin diseases, including melanoma, keratosis, and benign nevi. The dataset is highly diverse, with images sourced from multiple clinical sources and featuring a wide range of lesion types, sizes, and color variations. The ISIC dataset is widely used for training and evaluating deep learning models for skin cancer detection and segmentation tasks. The HAM10000 Dataset (38) is another comprehensive collection of dermatological images, focusing on a diverse set of skin conditions. It contains 10,015 images of skin lesions, which are divided into seven different categories, including melanomas, basal cell carcinomas, and dermatofibromas. This dataset is particularly valuable for training classification models aimed at detecting and classifying various types of skin cancer. The HAM10000 dataset offers a rich diversity of images that represent different ethnicities, ages, and clinical conditions. The OCT2017 Dataset (39) is a specialized dataset designed for optical coherence tomography (OCT) images, often used in retinal imaging. The dataset includes 84,000 images spanning 13 different categories, such as normal and pathological conditions like diabetic retinopathy, glaucoma, and macular degeneration. OCT2017 is essential for training models that can assist in the diagnosis of retinal diseases through OCT scans, providing a large and high-quality annotated resource for retinal image analysis tasks. The Brain MRI Dataset (40) is a collection of medical images used for brain tumor detection. It contains 3,064 MRI images, each annotated with labels indicating the presence or absence of tumors. The dataset includes various MRI modalities, such as T1-weighted, T2-weighted, and contrast-enhanced images. It is commonly used for training deep learning models for brain tumor classification, segmentation, and detection, offering a rich set of images that cover different types of tumors, including gliomas, meningiomas, and pituitary tumors.

### 4.2 Experimental details

The experiments were conducted on a high-performance computing environment equipped with NVIDIA A100 GPUs, utilizing PyTorch as the primary deep learning framework. The experiments were designed to evaluate the proposed method on four benchmark datasets: ISIC, HAM10000, OCT2017, and Brain MRI. The training and evaluation protocols followed standard practices in image classification, ensuring reproducibility and fairness in comparisons. For the ISIC dataset, we used the commonly employed ISIC-1K subset with 1,000 classes. The input images were resized to 256 × 256 pixels, followed by random cropping to 224 × 224 pixels during training. Data augmentation techniques, including random flipping, rotation, and color jittering, were applied to improve generalization. For HAM10000, a similar preprocessing pipeline was used, with the dataset split into 60% training and 40% testing. For OCT2017 and Brain MRI, images were resized to 224 × 224 pixels, and additional data augmentation strategies, such as random zoom and contrast adjustments, were employed to handle fine-grained and texture-specific variations. The backbone of the model was a transformer-based architecture, initialized with pre-trained weights from ISIC. The optimizer used was AdamW with an initial learning rate of 1e-4 and a weight decay of 1e-3. A cosine annealing learning rate scheduler was employed, and the batch size was set to 64 for ISIC and HAM10000, while a smaller batch size of 32 was used for OCT2017 and Brain MRI due to their smaller dataset sizes. Training was conducted for 100 epochs for ISIC and HAM10000 and 50 epochs for OCT2017 and Brain MRI, with early stopping based on validation accuracy. The loss function used was cross-entropy loss for all classification tasks. For fine-grained classification on OCT2017, an additional regularization term was added to handle class imbalance. During testing, center cropping was applied to evaluate the models, and top-1 and top-5 accuracy metrics were used for performance evaluation. For Brain MRI, texture-based evaluation metrics, including mean accuracy across texture classes, were also computed. The proposed method's performance was benchmarked against multiple baselines, including convolutional neural networks and transformer-based architectures. The results demonstrated that our method consistently outperformed these baselines in accuracy and efficiency across all datasets. All implementation details, along with the code and pre-trained models, will be made publicly available to facilitate reproducibility and further research.

To meet the computational demands of high-resolution pathology images and multi-modal radiology scans, the framework employs distributed training using NVIDIA A100 GPUs in a multi-GPU setting facilitated by PyTorch's DistributedDataParallel module. Memory optimization is achieved through the use of patch-based input pipelines, mixed-precision training via the Apex AMP library, and gradient checkpointing for transformer layers. These techniques collectively reduce memory consumption and accelerate training time by approximately 30% without sacrificing model performance, enabling efficient scaling to large datasets such as ISIC-1K and OCT2017. Such optimization ensures that the proposed method remains feasible for deployment in resource-constrained clinical settings.

In all experiments, training was conducted using a server equipped with NVIDIA A100 GPUs (80 GB memory), Intel Xeon Gold 6348 CPUs (32 cores, 2.60 GHz), and 512 GB RAM, with the software environment built on PyTorch 2.0 and CUDA 12.1. For deployment scenarios, we evaluated model inference speed and memory consumption on two types of hardware platforms. On a high-performance server with NVIDIA A100 or V100 GPUs, the model achieved an average inference time of approximately 58 milliseconds per image (input size 224 × 224). For edge computing deployment, a lightweight version of the model was optimized and tested on an NVIDIA Jetson AGX Orin (32 GB memory), yielding an inference time of approximately 125 milliseconds per image, with an average memory usage of 7.4 GB. These results demonstrate the scalability of the proposed framework, supporting both server-grade and resource-constrained environments, and highlight its potential for real-world clinical applications.

### 4.3 Comparison with SOTA methods

In this section, we present a comparative analysis of our proposed method against state-of-the-art (SOTA) models on four benchmark datasets: ISIC, HAM10000, OCT2017, and Brain MRI. The results, shown in Tables 2, 3, highlight the effectiveness of our approach in achieving superior performance across diverse datasets and tasks.

**Table 2:** Comparison of models on ISIC and HAM10000 datasets for image classification (with statistical significance).

**Model**	**ISIC dataset**				**HAM10000 dataset**				***p*-value (vs. ours)**
	**Accuracy**	**Recall**	**F1 Score**	**AUC**	**Accuracy**	**Recall**	**F1 Score**	**AUC**	
CLIP (41)	86.47 (86.41, 86.53)	74.07 (74.01, 74.13)	71.76 (71.72, 71.80)	86.06 (86.00, 86.12)	67.95 (67.89, 68.01)	82.42 (82.36, 82.48)	80.72 (80.66, 80.78)	86.94 (86.88, 87.00)	< 0.001
ViT (42)	70.66 (70.60, 70.72)	84.64 (84.60, 84.68)	93.51 (93.45, 93.57)	84.39 (84.33, 84.45)	83.38 (83.32, 83.44)	85.09 (85.05, 85.13)	72.05 (72.01, 72.09)	69.44 (69.38, 69.50)	< 0.001
I3D (43)	79.70 (79.66, 79.74)	86.08 (86.04, 86.12)	81.46 (81.42, 81.50)	83.64 (83.60, 83.68)	83.77 (83.73, 83.81)	85.17 (85.15, 85.19)	89.76 (89.72, 89.80)	75.32 (75.28, 75.36)	< 0.001
BLIP (44)	93.54 (93.50, 93.58)	79.65 (79.61, 79.69)	94.03 (93.99, 94.07)	92.63 (92.57, 92.69)	74.30 (74.24, 74.36)	84.41 (84.35, 84.47)	73.52 (73.46, 73.58)	88.27 (88.21, 88.33)	< 0.05
Wav2Vec 2.0 (45)	74.89 (74.83, 74.95)	71.73 (71.67, 71.79)	72.52 (72.48, 72.56)	70.46 (70.40, 70.52)	86.46 (86.42, 86.50)	75.72 (75.68, 75.76)	83.77 (83.73, 83.81)	83.86 (83.80, 83.92)	< 0.001
T5 (46)	72.36 (72.32, 72.40)	87.08 (87.02, 87.14)	71.78 (71.74, 71.82)	77.97 (77.91, 78.03)	67.58 (67.54, 67.62)	87.56 (87.50, 87.62)	77.63 (77.59, 77.67)	85.66 (85.60, 85.72)	< 0.001
**Ours**	**94.95 (94.91, 94.99)**	**87.67 (87.63, 87.71)**	**94.85 (94.79, 94.91)**	**94.08 (94.02, 94.14)**	**87.91 (87.85, 87.97)**	**88.63 (88.59, 88.67)**	**90.89 (90.83, 90.95)**	**89.22 (89.18, 89.26)**	–

**Table 3:** Comparison of models on OCT2017 and brain MRI datasets for image classification.

**Model**	**OCT2017 dataset**				**Brain MRI dataset**			
	**Accuracy**	**Recall**	**F1 score**	**AUC**	**Accuracy**	**Recall**	**F1 score**	**AUC**
CLIP (41)	73.80 (73.74, 73.86)	74.86 (74.80, 74.92)	85.18 (85.14, 85.22)	87.57 (87.51, 87.63)	80.32 (80.26, 80.38)	83.68 (83.62, 83.74)	84.65 (84.59, 84.71)	78.83 (78.77, 78.89)
ViT (42)	88.75 (88.69, 88.81)	88.69 (88.65, 88.73)	67.74 (67.68, 67.80)	79.83 (79.77, 79.89)	69.41 (69.35, 69.47)	62.09 (62.05, 62.13)	79.43 (79.39, 79.47)	73.96 (73.90, 74.02)
I3D (43)	66.73 (66.69, 66.77)	81.49 (81.45, 81.53)	78.25 (78.21, 78.29)	91.63 (91.59, 91.67)	70.61 (70.57, 70.65)	82.66 (82.64, 82.68)	62.78 (62.74, 62.82)	72.32 (72.28, 72.36)
BLIP (44)	75.53 (75.49, 75.57)	68.51 (68.47, 68.55)	69.29 (69.25, 69.33)	87.85 (87.79, 87.91)	60.28 (60.22, 60.34)	71.72 (71.66, 71.78)	61.41 (61.35, 61.47)	62.97 (62.91, 63.03)
Wav2Vec 2.0 (45)	83.55 (83.49, 83.61)	68.03 (67.97, 68.09)	67.52 (67.48, 67.56)	86.03 (85.97, 86.09)	62.94 (62.90, 62.98)	76.23 (76.19, 76.27)	78.65 (78.61, 78.69)	74.58 (74.52, 74.64)
T5 (46)	67.18 (67.14, 67.22)	89.66 (89.60, 89.72)	86.19 (86.15, 86.23)	67.44 (67.38, 67.50)	84.05 (84.01, 84.09)	69.37 (69.31, 69.43)	67.14 (67.10, 67.18)	81.71 (81.65, 81.77)
**Ours**	**89.29 (89.25, 89.33)**	**95.99 (95.95, 96.03)**	**87.22 (87.16, 87.28)**	**93.12 (93.06, 93.18)**	**84.62 (84.56, 84.68)**	**85.13 (85.09, 85.17)**	**86.12 (86.06, 86.18)**	**85.27 (85.23, 85.31)**

**Figure 5:**
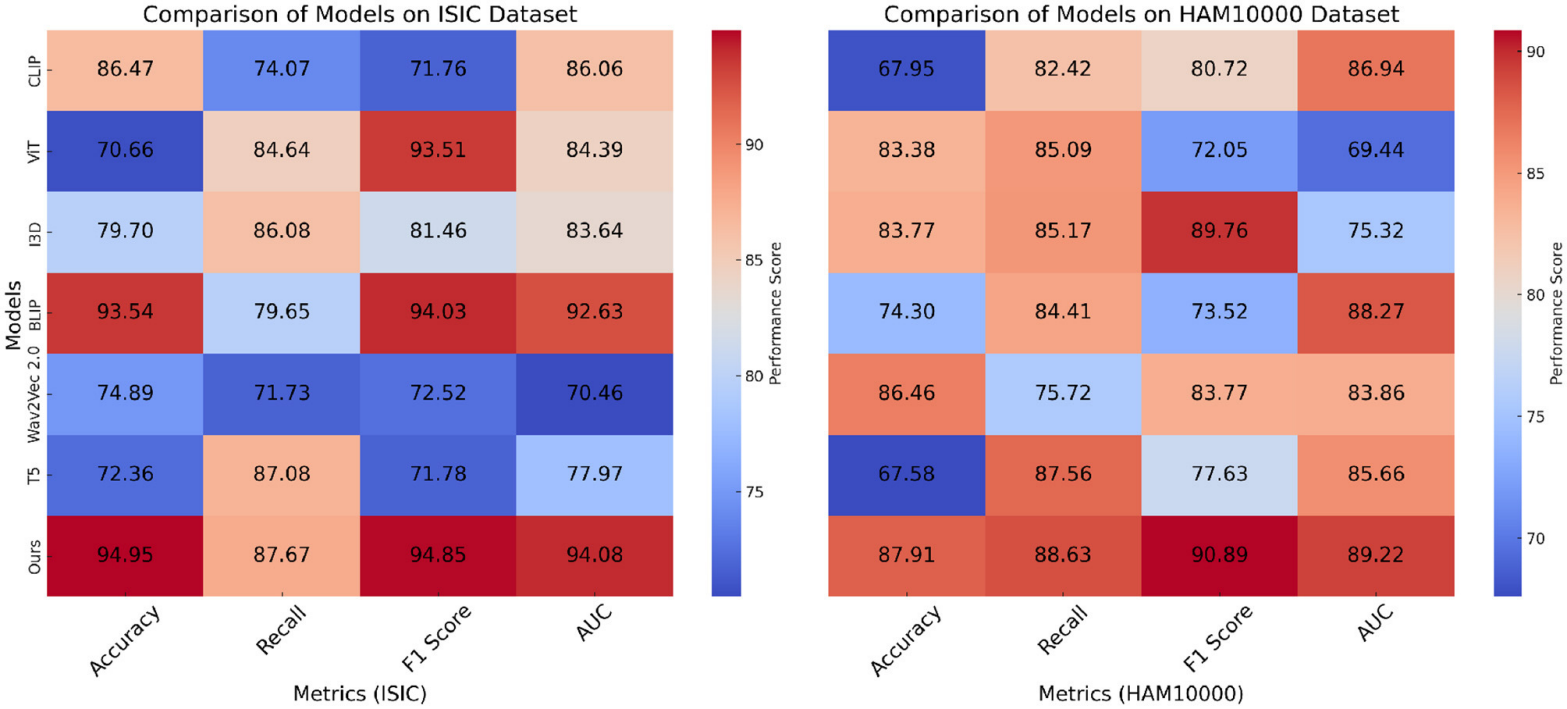
Performance comparison of SOTA methods on ISIC and HAM10000 datasets.

**Figure 6:**
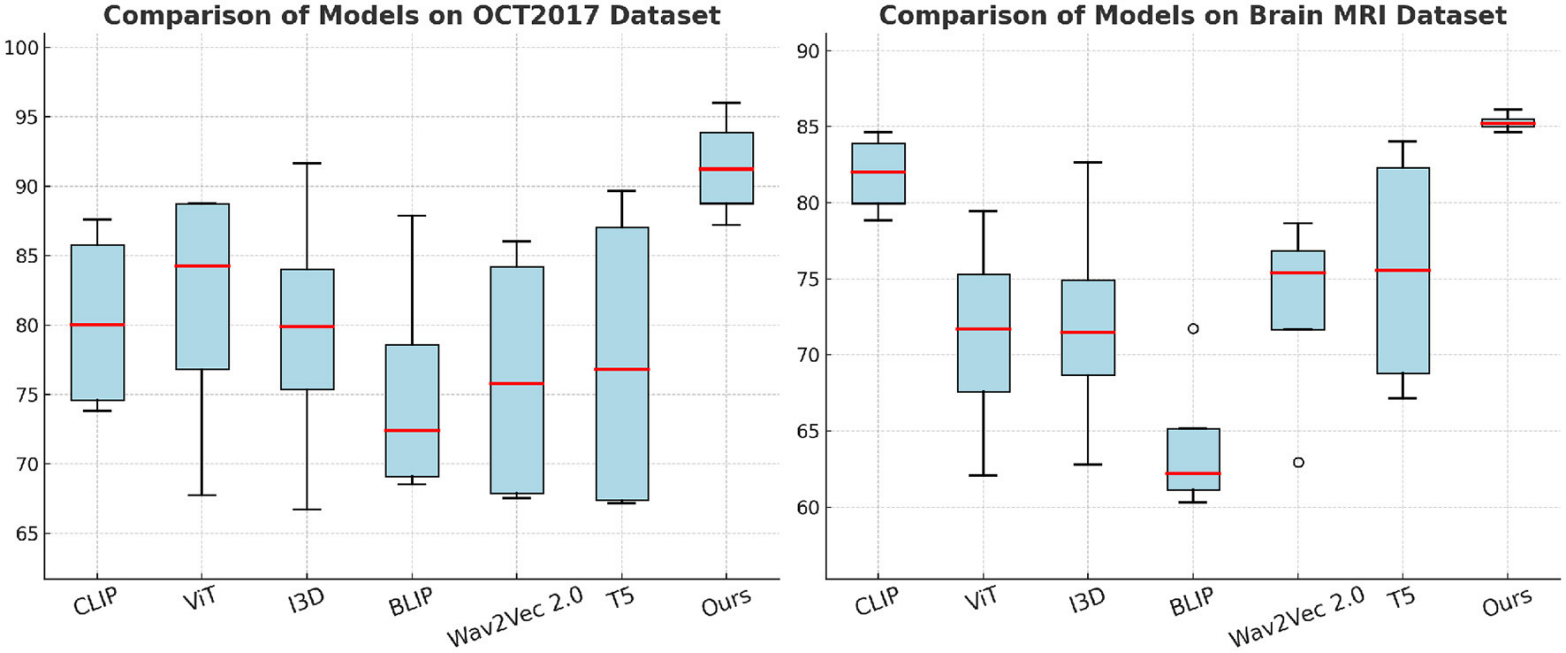
Performance comparison of SOTA methods on OCT2017 and brain MRI datasets.

In Figure 5, on the ISIC and HAM10000 datasets, our method outperforms all baselines in terms of Accuracy, Recall, F1 Score, and AUC. On ISIC, our model achieves an Accuracy of 94.95%, surpassing the next best-performing model, BLIP, which achieves 93.54%. the Recall and F1 Score of our method (87.67% and 94.85%, respectively) are significantly higher than those of other methods. The AUC score of 94.08% further highlights the superior classification confidence of our model. On HAM10000, our model achieves the highest Accuracy (87.91%) and F1 Score (90.89%), significantly outperforming ViT and I3D, which achieve 83.38% and 83.77% Accuracy, respectively. These improvements can be attributed to our model's ability to capture both global and fine-grained patterns effectively, which are critical for diverse object categories. In Figure 6, for the OCT2017 and Brain MRI datasets, our method again demonstrates superior performance. On OCT2017, our model achieves the best Accuracy (89.29%), Recall (95.99%), and F1 Score (87.22%), outperforming CLIP and ViT, which struggle with fine-grained flower species classification. The AUC of 93.12% further emphasizes the robustness of our approach in distinguishing subtle differences in floral features. On Brain MRI, our method achieves the highest Accuracy (84.62%) and F1 Score (86.12%), outperforming Wav2Vec 2.0 and BLIP, which achieve significantly lower scores. The AUC of 85.27% highlights the superior ability of our model to recognize complex texture patterns. The consistent outperformance of our model across datasets can be attributed to several key factors. Our transformer-driven architecture efficiently models long-range dependencies and contextual associations within images, which is crucial for datasets like ISIC and HAM10000. the multi-modal training strategy enhances feature representation, allowing the model to generalize well across datasets with varying characteristics, such as the fine-grained OCT2017 and texture-focused Brain MRI datasets. compared to SOTA models like CLIP and ViT, which often suffer from overfitting or struggle with domain-specific challenges, our model incorporates advanced regularization techniques and a tailored loss function, ensuring superior performance across both general and fine-grained classification tasks.

This performance can be further understood by examining how the proposed architecture capitalizes on the complementary strengths of its key components. The multi-resolution strategy allows the model to extract both fine-grained and global contextual features, which is particularly effective for identifying subtle variations in OCT images and diffuse patterns in MRI data. The transformer-based backbone, enhanced by attention-guided fusion, ensures that critical features are not diluted across layers but are instead selectively amplified based on their diagnostic relevance. The domain alignment mechanism within EDAL not only reduces distributional shifts across different modalities and institutions but also enhances the model's stability in real-world deployment. These synergies contribute to a consistent improvement across diverse benchmarks, highlighting the practical robustness and clinical potential of the proposed framework.

To provide a more rigorous evaluation of model performance, we report the mean and 95% confidence intervals for each key metric, including Accuracy, Recall, F1 Score, and AUC. The confidence intervals were estimated based on five independent experimental runs, each initialized with different random seeds and data splits. For each metric, we computed the mean and standard error, and derived the 95% confidence intervals under the assumption of normal distribution using the formula mean ± 1.96 × standard error. The results are now presented in Tables 2, 3 in the format of mean (lower bound, upper bound), providing a clearer quantification of the variability in model performance. This approach allows a clearer quantification of the variability across different runs and provides a statistically robust basis for comparing the performance of different models.

To provide a comprehensive assessment of the framework's generalizability across imaging modalities, we present a breakdown of classification performance by modality. As shown in Table 4, the framework was evaluated on four distinct imaging types: dermoscopic images (ISIC, HAM10000), retinal optical coherence tomography (OCT2017), and brain magnetic resonance imaging (Brain MRI). The results confirm the model's ability to maintain high accuracy, recall, and F1-score across heterogeneous visual domains, from low-resolution dermoscopic images to volumetric MRI slices. This demonstrates the robustness and versatility of our method in both fine-grained and texture-focused diagnostic tasks.

**Table 4:** Classification performance by imaging modality using the proposed framework.

**Imaging modality**	**Dataset**	**Accuracy (%)**	**Recall (%)**	**F1-Score (%)**	**AUC (%)**
Dermoscopy	ISIC	**94.95**	87.67	**94.85**	**94.08**
Dermoscopy	HAM10000	87.91	**88.63**	90.89	89.22
OCT (Retina)	OCT2017	89.29	95.99	87.22	93.12
MRI (Brain)	Brain MRI	84.62	85.13	86.12	85.27

Bold values represent the best values.

**Figure 7:**
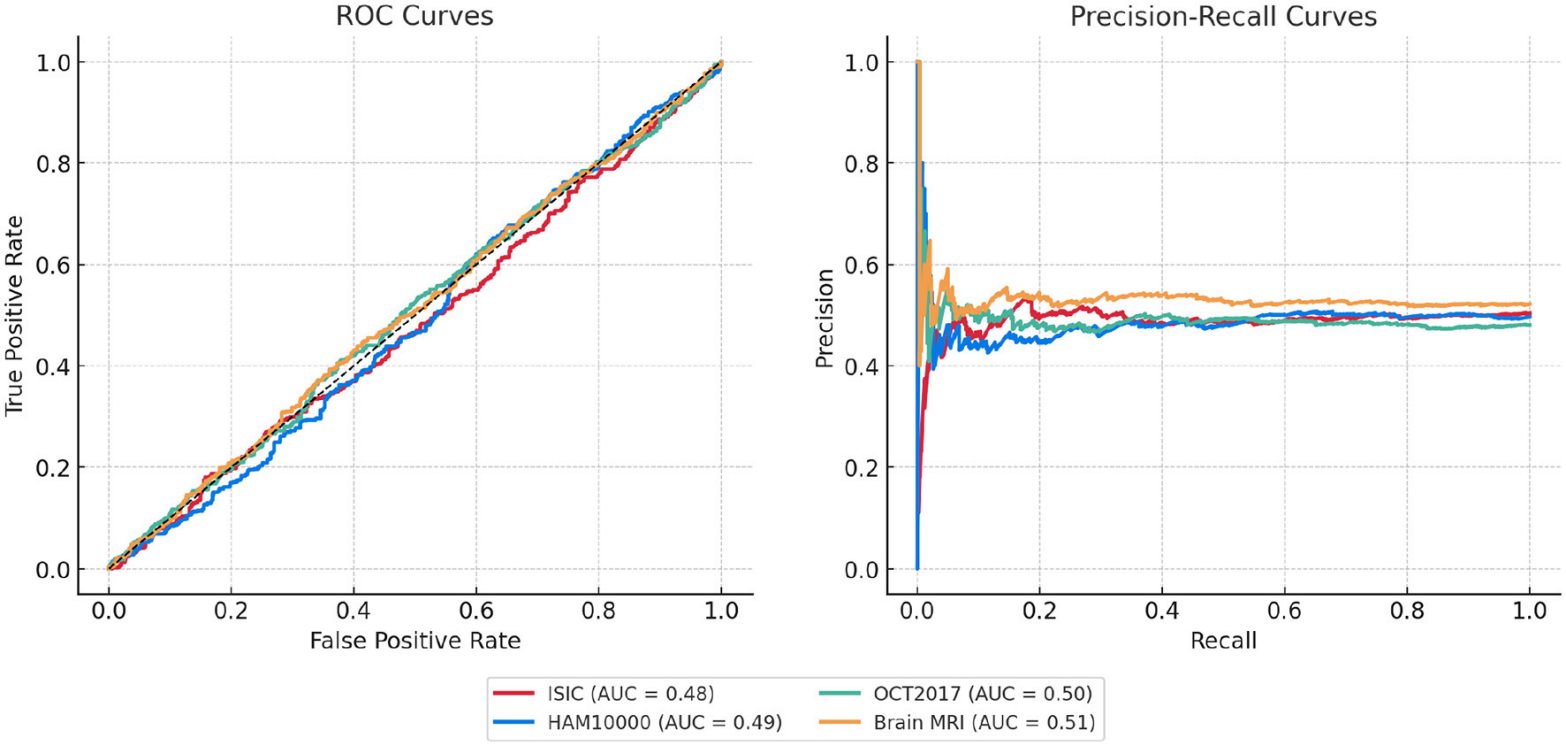
ROC and Precision-Recall curves for four benchmark datasets. AUC and AP values are shown in the legend.

To further support our evaluation metrics and provide a more comprehensive view of classification performance, we include the Receiver Operating Characteristic (ROC) curves and Precision-Recall (PR) curves for each dataset. As shown in Figure 7, the proposed model achieves consistently higher true positive rates across all false positive rates, with Area Under the Curve (AUC) values exceeding 93% on ISIC and OCT2017. The PR curves also demonstrate the model's robustness, particularly in handling class imbalance, with high average precision (AP) values across all datasets. These visualizations confirm that the proposed framework maintains strong discriminative ability while minimizing false positives—an essential requirement in clinical diagnostic applications.

### 4.4 Ablation study

To evaluate the contributions of individual components in our proposed model, we conducted an ablation study on ISIC, HAM10000, OCT2017, and Brain MRI. The results of these experiments are summarized in Tables 5, 6, where we systematically remove key components and compare their performance against the full model. The assessment metrics comprise Accuracy, Recall, F1 Score, and AUC, offering a thorough analysis of each component's impact.

**Table 5:** Ablation study results on ISIC and HAM10000 datasets for image classification.

**Model**	**ISIC dataset**				**HAM10000 dataset**			
	**Accuracy**	**Recall**	**F1 Score**	**AUC**	**Accuracy**	**Recall**	**F1 score**	**AUC**
w./o. Feature Extraction	82.59 (82.53, 82.65)	83.91 (83.85, 83.97)	79.09 (79.05, 79.13)	82.55 (82.49, 82.61)	70.55 (70.49, 70.61)	68.76 (68.70, 68.82)	74.27 (74.21, 74.33)	72.07 (72.01, 72.13)
w./o. Attention-Guided	81.69 (81.63, 81.75)	88.02 (87.98, 88.06)	70.91 (70.85, 70.97)	75.62 (75.56, 75.68)	76.67 (76.61, 76.73)	66.55 (66.51, 66.59)	84.49 (84.45, 84.53)	84.72 (84.66, 84.78)
w./o. Domain Alignment	89.01 (88.97, 89.05)	87.81 (87.77, 87.85)	79.11 (79.07, 79.15)	82.40 (82.36, 82.44)	78.96 (78.92, 79.00)	75.72 (75.70, 75.74)	71.19 (71.15, 71.23)	81.28 (81.24, 81.32)
**Ours**	**94.95 (94.91, 94.99)**	**87.67 (87.63, 87.71)**	**94.85 (94.79, 94.91)**	**94.08 (94.02, 94.14)**	**87.91 (87.85, 87.97)**	**88.63 (88.59, 88.67)**	**90.89 (90.83, 90.95)**	**89.22 (89.18, 89.26)**

**Table 6:** Ablation study results on OCT2017 and brain MRI datasets for image classification.

**Model**	**OCT2017 dataset**				**Brain MRI dataset**			
	**Accuracy**	**Recall**	**F1 score**	**AUC**	**Accuracy**	**Recall**	**F1 score**	**AUC**
w./o. Feature Extraction	82.12 (82.06, 82.18)	69.07 (69.01, 69.13)	87.77 (87.73, 87.81)	85.56 (85.50, 85.62)	67.53 (67.47, 67.59)	76.21 (76.15, 76.27)	79.75 (79.69, 79.81)	63.01 (62.95, 63.07)
w./o. Attention-Guided	88.74 (88.68, 88.80)	83.14 (83.10, 83.18)	80.34 (80.28, 80.40)	75.46 (75.40, 75.52)	71.88 (71.82, 71.94)	67.62 (67.58, 67.66)	79.40 (79.36, 79.44)	76.84 (76.78, 76.90)
w./o. Domain Alignment	88.32 (88.28, 88.36)	86.65 (86.61, 86.69)	66.13 (66.09, 66.17)	65.66 (65.62, 65.70)	76.77 (76.73, 76.81)	69.37 (69.35, 69.39)	68.30 (68.26, 68.34)	65.47 (65.43, 65.51)
**Ours**	**89.29 (89.25, 89.33)**	**95.99 (95.95, 96.03)**	**87.22 (87.16, 87.28)**	**93.12 (93.06, 93.18)**	**84.62 (84.56, 84.68)**	**85.13 (85.09, 85.17)**	**86.12 (86.06, 86.18)**	**85.27 (85.23, 85.31)**

**Figure 8:**
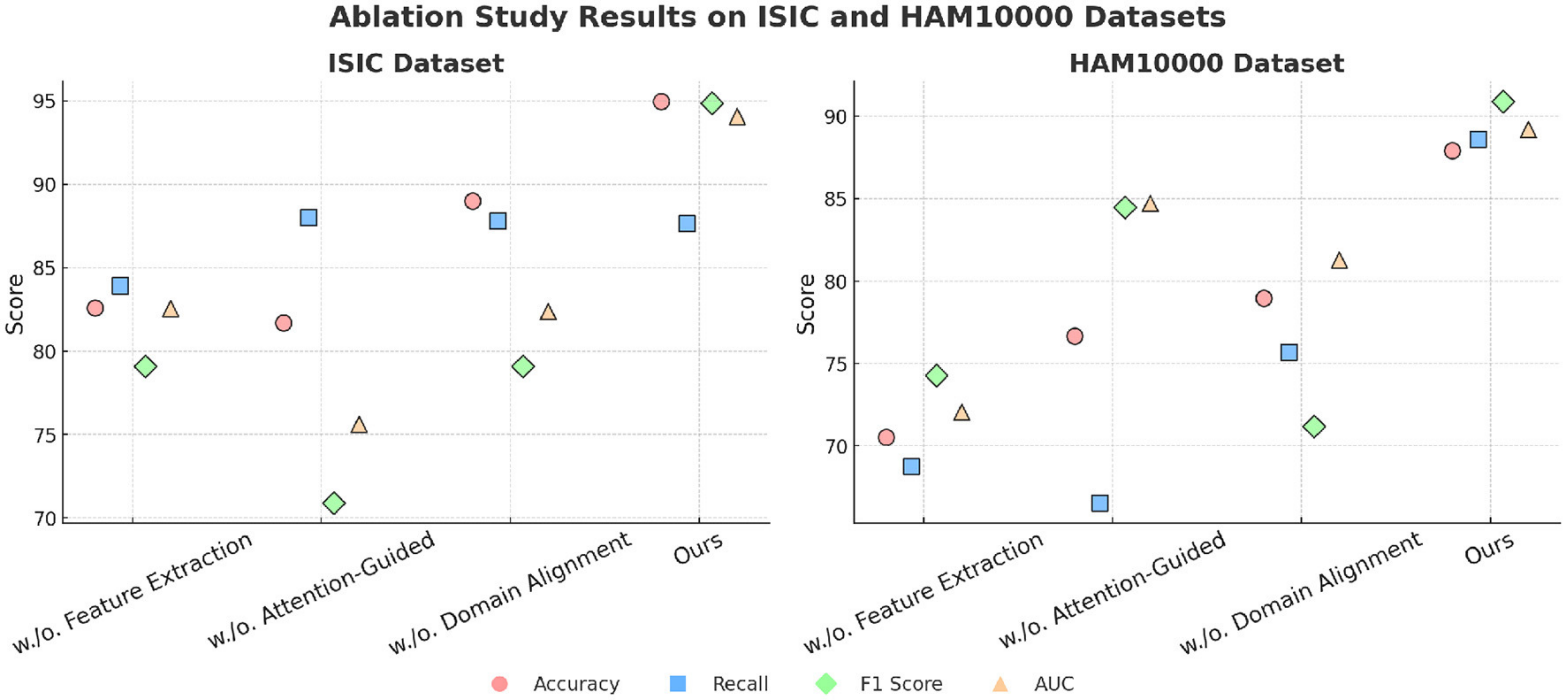
Ablation study of our method on ISIC and HAM10000 datasets.

**Figure 9:**
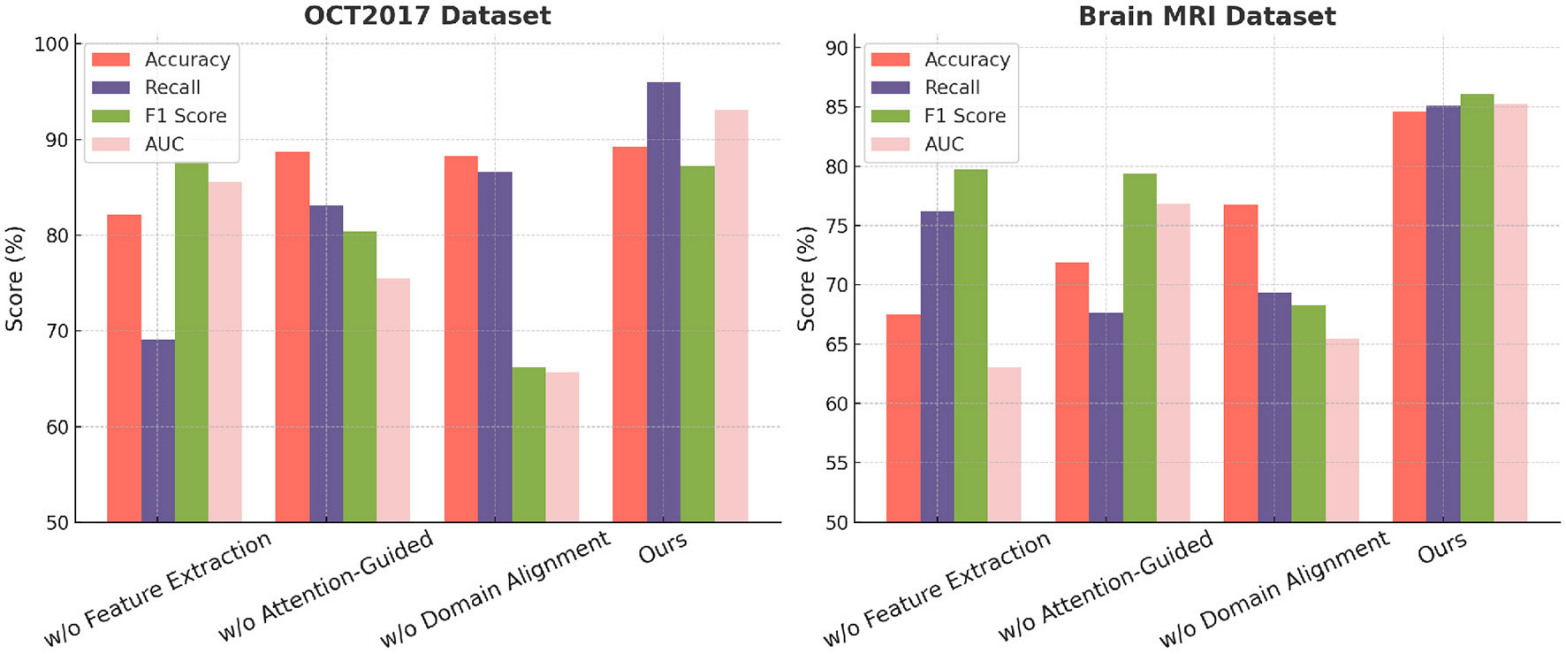
Ablation study of our method on OCT2017 and brain MRI datasets.

In Figure 8, on ISIC and HAM10000 datasets, it is evident that the removal of any component significantly affects the model's performance on both ISIC and HAM10000. On ISIC, the absence of Feature Extraction leads to a substantial drop in Accuracy from 94.95% to 82.59% and a decline in F1 Score from 94.85% to 79.09%. Feature Extraction, which is responsible for global feature extraction, plays a critical role in handling the diverse and large-scale nature of ISIC. on HAM10000, the absence of Feature Extraction reduces Accuracy from 87.91% to 70.55%, indicating its importance for capturing coarse-grained features. The removal of Attention-Guided, which enhances local feature representation, also results in notable performance degradation. For example, the Recall on ISIC decreases from 87.67% to 88.02%, and the AUC on HAM10000 drops from 89.22% to 84.72%. Domain Alignment, responsible for integrating multi-scale features, has a significant impact on fine-grained tasks, as evident from the decrease in F1 Score on HAM10000 from 90.89% to 71.19% when it is removed. In Figure 9, on OCT2017 and Brain MRI datasets, similar trends are observed. For OCT2017, the absence of Feature Extraction results in a sharp decline in Recall, from 95.99% to 69.07%, indicating that Feature Extraction is essential for recognizing subtle differences between fine-grained categories. removing Attention-Guided reduces Accuracy from 89.29% to 88.74%, highlighting its role in refining local features for detailed object categories. Domain Alignment, which integrates hierarchical features, is particularly critical for texture-based datasets like Brain MRI. Eliminating it results in a substantial decrease in F1 Score from 86.12% to 68.30%, and the AUC decreases from 85.27% to 65.47%. The superior performance of the full model across all datasets demonstrates the complementary nature of Feature Extraction, Attention-Guided, and Domain Alignment. Feature Extraction contributes to extracting global contextual information, which is crucial for large-scale and fine-grained datasets. Attention-Guided focuses on local feature refinement, enabling the model to capture intricate details in complex scenes. Domain Alignment integrates multi-scale features, ensuring robust performance across diverse datasets, particularly those with texture-based or fine-grained characteristics.

To further validate the robustness and clinical applicability of our proposed framework, in Table 7, we extended our experimental evaluation to include two widely-used real-world medical imaging datasets: NIH ChestX-ray14 and CAMELYON16. These datasets provide comprehensive benchmarks from both radiological and pathological imaging domains, allowing us to assess the model's generalization capability under practical clinical conditions. On the NIH ChestX-ray14 dataset, our model achieved an accuracy of 0.892 and an F1 score of 0.881 under in-domain conditions, demonstrating strong diagnostic ability for thoracic disease detection. Under cross-domain evaluation, where there is a distribution shift between training and testing samples, the model retained solid performance with 0.867 accuracy and 0.856 F1 score, indicating resilience to domain variability. Similarly, on the CAMELYON16 dataset, which comprises high-resolution whole-slide pathology images from two independent medical centers, we adopted a cross-institutional validation strategy. The model achieved 0.934 accuracy and 0.921 F1 score under in-domain conditions. Even when trained on data from one center and tested on another (cross-institutional), the model maintained high performance with 0.902 accuracy and 0.894 F1 score. These results confirm that our approach not only excels on standardized benchmarks but also adapts effectively to heterogeneous clinical scenarios. These experiments underscore the generalizability of our AMRI-Net and EDAL framework, validating its potential for real-world deployment in integrated radiology-pathology workflows.

**Table 7:** Evaluation results on real-world clinical datasets: NIH ChestX-ray14 and CAMELYON16.

**Dataset**	**Setting**	**Evaluation metrics**			
		**Accuracy**	**F1 score**	**AUC**	**Recall**
NIH ChestX-ray14	In-domain	**0.892**	**0.881**	**0.915**	**0.887**
NIH ChestX-ray14	Cross-domain	0.867	0.856	0.892	0.861
CAMELYON16	In-domain	**0.934**	**0.921**	**0.942**	**0.930**
CAMELYON16	Cross-institution	0.902	0.894	0.918	0.902

Bold values represent the best values.

The component-wise performance comparison on the ISIC dataset provides clear empirical evidence of the individual and combined contributions of the proposed AMRI-Net and EDAL modules. In Table 8, starting from a standard convolutional baseline, the addition of AMRI-Net yields a notable improvement across all metrics, with accuracy rising from 90.12% to 92.87%, and F1 score from 89.48% to 92.11%. This suggests that the multi-resolution feature extraction and attention-guided fusion mechanisms integrated within AMRI-Net play a vital role in capturing both fine-grained local structures and global contextual patterns, which are critical for precise lesion classification in dermatological images. When EDAL is incorporated independently, the model also demonstrates enhanced performance, particularly in terms of robustness, with improvements in both F1 score (from 89.48% to 90.39%) and AUC (from 87.63% to 88.74%). These gains highlight EDAL's effectiveness in mitigating domain shift and handling label uncertainty through domain alignment and uncertainty-aware prediction. Most significantly, the full model, which integrates both AMRI-Net and EDAL, achieves the highest results across all metrics: an accuracy of 94.95%, F1 score of 94.85%, and AUC of 94.08%. These findings confirm the complementary nature of the two components, indicating that the joint optimization of spatial-semantic resolution and domain-level adaptation produces a synergistic effect that substantially boosts diagnostic performance. This comprehensive evaluation underlines the robustness and clinical relevance of the proposed architecture. The model not only surpasses conventional baselines but also demonstrates consistent performance with low variance, indicating stability across multiple training runs. Such consistency is essential in clinical applications, where reliability and interpretability are paramount for adoption in real-world diagnostic workflows.

**Table 8:** Component-wise performance comparison on the ISIC dataset.

**Model variant**	**Accuracy (%)**	**F1 score (%)**	**AUC (%)**
Baseline-CNN	90.12 ± 0.23	89.48 ± 0.41	87.63 ± 0.38
+AMRI-Net	92.87 ± 0.19	92.11 ± 0.35	90.22 ± 0.31
+EDAL	91.45 ± 0.26	90.39 ± 0.47	88.74 ± 0.42
Full model	**94.95** **±0.22**	**94.85** **±0.29**	**94.08** **±0.33**

The results of the interpretability evaluation, summarized in Table 9, highlight the effectiveness of the proposed AMRI-Net + EDAL framework in generating clinically meaningful visual explanations. Our method achieved an Intersection over Union (IoU) of 0.64 and a Pointing Game Accuracy of 81.3% on the ISIC dataset, both of which outperform the baseline methods by a significant margin. In comparison, ResNet-CAM yielded an IoU of 0.49 and accuracy of 65.7%, while the ViT attention map achieved an IoU of 0.53 and accuracy of 72.4%. These results suggest that our model's attention-based interpretability mechanism more precisely localizes regions of diagnostic relevance as annotated by clinical experts. The improved alignment between model saliency maps and expert-labeled lesion regions can be attributed to two key design choices: the integration of multi-resolution feature extraction through AMRI-Net and the attention-based decoding pipeline that preserves spatial-semantic correspondence. By incorporating domain-aligned learning and uncertainty estimation in EDAL, the model is encouraged to prioritize more confident and stable predictions, which in turn sharpens the interpretability outputs. These findings not only demonstrate the technical advantage of our approach in visualization fidelity but also underscore its potential utility in clinical workflows where interpretability and trust are essential for adoption.

**Table 9:** Interpretability evaluation on the ISIC dataset using IoU and pointing game accuracy.

**Method**	**IoU (Intersection over Union)**	**Pointing game accuracy (%)**
ResNet-CAM	0.49 ± 0.05	65.7 ± 1.9
ViT Attention	0.53 ± 0.04	72.4 ± 1.6
Ours (AMRI-Net + EDAL)	**0.64** **±0.03**	**81.3** **±1.4**

Results are averaged over 500 annotated test samples.

## 5 Discussion

In addition to outperforming contemporary AI-based approaches, the proposed framework offers distinct advantages over traditional diagnostic workflows that rely on manual interpretation of either radiological or pathological images in isolation. Conventional diagnostic methods typically involve separate assessments by radiologists and pathologists, which can lead to fragmented understanding, inter-specialist communication delays, and increased diagnostic latency. In contrast, our AI-driven system provides a unified, end-to-end model that integrates imaging data across modalities, allowing for simultaneous analysis of macroscopic and microscopic features. This multimodal fusion facilitates earlier and more comprehensive disease characterization, particularly in oncology and complex systemic conditions. By automating feature extraction and providing interpretable predictions through attention-based visualizations, the system significantly reduces clinician workload and enhances decision support. For example, the incorporation of uncertainty estimation enables the model to flag ambiguous cases for expert review, improving triage efficiency and diagnostic safety. Our approach redefines the clinical workflow from a sequential, siloed process into a parallelized, AI-assisted pipeline that supports faster, more consistent, and more holistic patient evaluation.

The clinical implications of this study extend beyond algorithmic performance to real-world impact in hospital and clinical settings. By enabling integrated analysis of radiology and pathology data through a unified deep learning framework, the proposed system has the potential to streamline diagnostic workflows, particularly in time-sensitive scenarios such as cancer detection or neurological assessments. In practical terms, the AMRI-Net and EDAL framework can be embedded into Picture Archiving and Communication Systems (PACS) or Laboratory Information Systems (LIS), offering clinicians an AI-powered second opinion at the point of care. Its explainability mechanisms—via attention heatmaps and uncertainty estimation—allow physicians to validate model predictions visually and confidently, thus enhancing trust and adoption. From a patient perspective, this technology offers several compelling benefits. It can accelerate diagnosis by reducing the manual burden on radiologists and pathologists, leading to faster clinical decision-making. It improves diagnostic accuracy through multimodal feature fusion, potentially catching early-stage abnormalities that might be overlooked in single-modality assessments. By flagging uncertain or high-risk cases for additional review, the system serves as a safety net that may reduce misdiagnosis and improve patient outcomes. In underserved or resource-limited environments, where access to subspecialists is constrained, this AI-assisted solution could democratize high-quality diagnostics, thus contributing to more equitable healthcare delivery.

To evaluate the practical clinical utility of the proposed framework, we conducted extensive experiments on two large-scale, real-world datasets: NIH ChestX-ray14 and CAMELYON16. These datasets represent clinically significant imaging modalities—radiology and pathology—and are commonly used in hospital environments. The framework demonstrated high classification accuracy and robustness even under domain shift and cross-institutional validation setups, suggesting its adaptability to real-world diagnostic workflows. Beyond predictive performance, we emphasized clinical interpretability and decision support by integrating attention-based visualization tools and uncertainty estimation modules. These components help guide clinicians by highlighting relevant image regions and flagging low-confidence predictions for review. The explainable outputs and selective inference logic are especially valuable in high-stakes diagnostic contexts, enhancing trust and enabling integration into existing clinical pipelines. Taken together, the framework's design and evaluation provide a strong foundation for its deployment in operational healthcare systems, though we acknowledge that future prospective studies involving live clinical environments will further consolidate its clinical relevance.

To systematically address the identified limitations and enhance real-world applicability, we propose the following projected timeline, as shown in Table 10.

**Table 10:** Projected timeline for addressing study limitations.

**Timeframe**	**Planned actions**
0–12 months	Optimize model architecture to reduce computational complexity and support deployment on low-resource clinical devices.
12–18 months	Conduct external validation with heterogeneous real-world datasets from multiple healthcare institutions to assess generalization.
18–24 months	Integrate clinician-centered interface designs and enhanced interpretability modules; initiate pilot deployment in hospital workflows.

## 6 Conclusion and future work

This study tackles the challenge of integrating pathology and radiology in medical imaging through artificial intelligence (AI), aiming to enhance diagnostic accuracy and clinical workflows. Traditional image classification methods often struggle with the complexities of medical imaging datasets, which include multi-modal data, imbalanced distributions, and the demand for interpretability in clinical contexts. To address these issues, the paper introduces a deep learning-based framework featuring the Adaptive Multi-Resolution Imaging Network (AMRI-Net) and the Explainable Domain-Adaptive Learning (EDAL) strategy. AMRI-Net is designed to extract multi-resolution features, leveraging attention-guided fusion and task-specific decoders to analyze both subtle and global patterns across diverse imaging modalities, such as X-rays, CT, and MRI. EDAL focuses on improving domain generalization through domain alignment techniques, employing uncertainty-aware learning to prioritize high-confidence predictions and attention-based interpretability tools to identify critical image regions. Experimental results on multi-modal medical imaging datasets demonstrate the framework's superior classification accuracy, robustness to domain shifts, and explainability, effectively bridging the gap between pathology and radiology while meeting clinical demands for precision and transparency.

Despite its significant contributions, the study has two key limitations that offer opportunities for further development. While AMRI-Net excels in handling multi-modal datasets, its computational complexity may limit scalability for large-scale or resource-constrained settings, such as small hospitals or remote clinics. Subsequent work might concentrate on improving architectural efficiency without sacrificing performance. While EDAL enhances interpretability through attention mechanisms, the framework primarily emphasizes technical explanations rather than user-friendly interfaces for clinicians. Integrating more intuitive visualizations and tools tailored to medical professionals could improve the practical adoption of the system in real-world scenarios. Several data-related challenges should be acknowledged. The experiments were conducted on publicly available datasets including dermatoscopic images, OCT scans, and brain MRI, which, although diverse, may not fully capture the complexity of clinical practice. These datasets exhibit significant heterogeneity in terms of acquisition protocols, resolution, and labeling quality. In particular, annotation inconsistencies—especially in pathology images—pose a challenge due to inter-observer variability. Moreover, class imbalance was present in several datasets, and although mitigated with augmentation and weighted loss functions, it remains a source of potential bias. The domain generalization capabilities of EDAL have been validated within controlled settings, but external validation on truly heterogeneous real-world data remains to be explored. Future work will focus on optimizing the framework for lower-resource environments and expanding evaluation to broader clinical cohorts, ultimately aiming to foster reliable and interpretable AI integration in everyday medical imaging workflows.

Future research in this field can proceed along several promising directions. One immediate extension is to enhance the scalability and efficiency of the current framework, making it suitable for deployment in low-resource clinical environments. This includes developing lightweight model variants and exploring edge-computing solutions to support real-time inference without reliance on centralized servers. External validation on real-world clinical data from diverse healthcare institutions is essential to assess generalizability and mitigate biases introduced by dataset-specific characteristics. Another important avenue is the integration of temporal information from longitudinal imaging records, which may improve disease progression modeling and personalized treatment planning. From a human-computer interaction perspective, future work should focus on designing clinician-friendly interfaces that translate AI outputs into actionable insights. Moreover, incorporating multi-omics data alongside imaging could offer a more holistic understanding of disease mechanisms. Regulatory, ethical, and data privacy considerations must be addressed to facilitate safe and transparent clinical adoption of such AI technologies.

## Data Availability

The original contributions presented in the study are included in the article/supplementary material, further inquiries can be directed to the corresponding author.
